# Supplementary material for: Characterization of GRK5 as a novel regulator of rhabdomyosarcoma tumor cell growth and self-renewal
Source: Oncotarget. 2020 Apr 21;11(16):1448–61. doi: 10.18632/oncotarget.27562 (PMC7185065; doi:10.18632/oncotarget.27562)
Supplement: Supplementary file 1 [file oncotarget-11-1448-s001.pdf]

Characterization of GRK5 as a novel regulator of rhabdomyosarcoma tumor cell growth and self-renewal

SUPPLEMENTARY MATERIALS

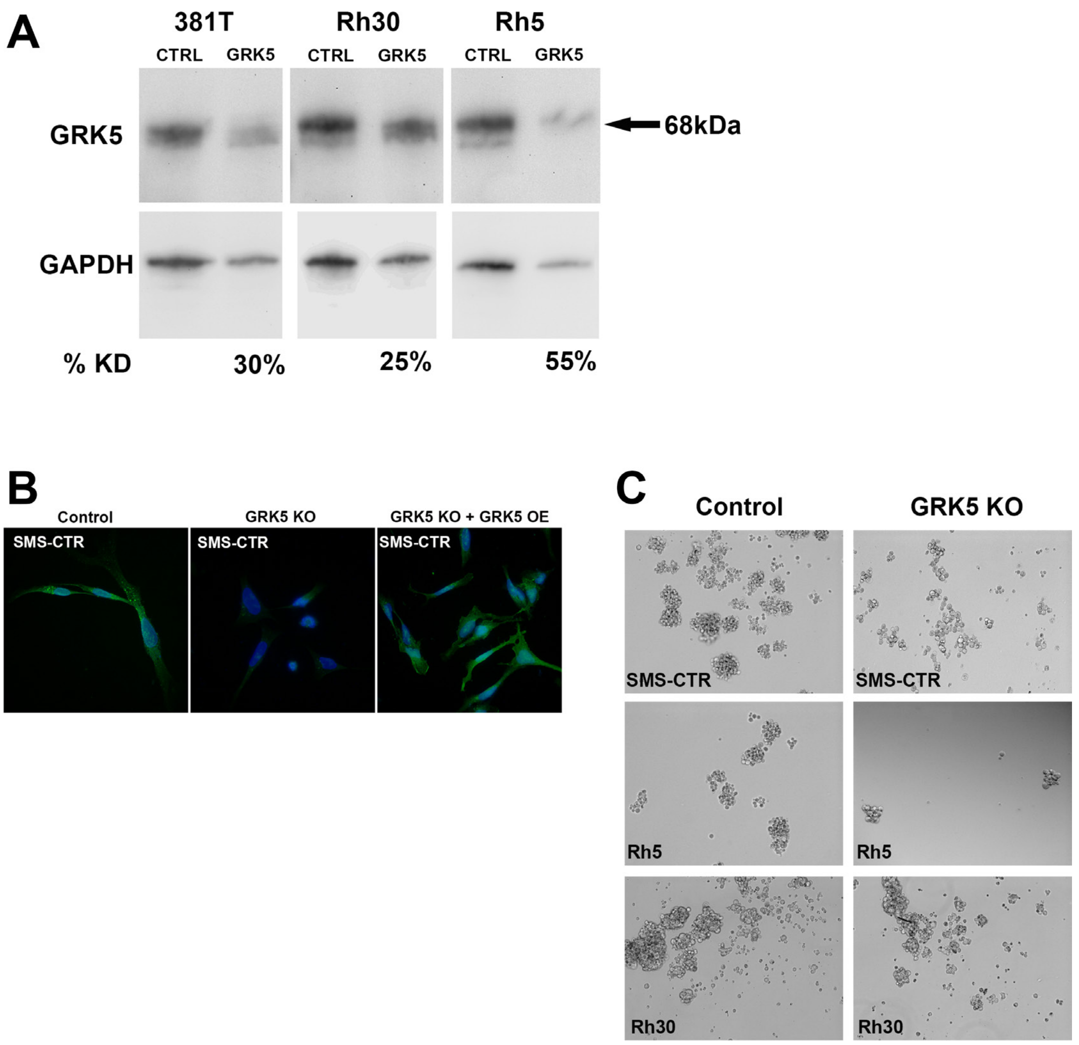

Supplementary Figure 1: Confirmation of GRK5 knockdown and spheroid images. (**A**) Western analysis demonstrating GRK5 protein depletion in a panel of RMS cell lines (381T, Rh30, Rh5) with Cas9-mediated targeted disruption of *GRK5*. (**B**) Immunofluorescence images demonstrating the specificity of the GRK5 antibody. SMS-CTR cells with knockout (GRK5 KO) shows reduced staining compared to Cas9-only control (CTRL) cells and the expression was restored in *GRK5* knockout cells expressing Cas9-resistant wild-type GRK5 (GRK5 KO+GRK5 OE). (**C**) Representative images of spheroid formation comparing control (CTRL) and GRK5 knockout (*GRK5* KO) in RMS cell lines (SMS-CTR, Rh5, Rh30).

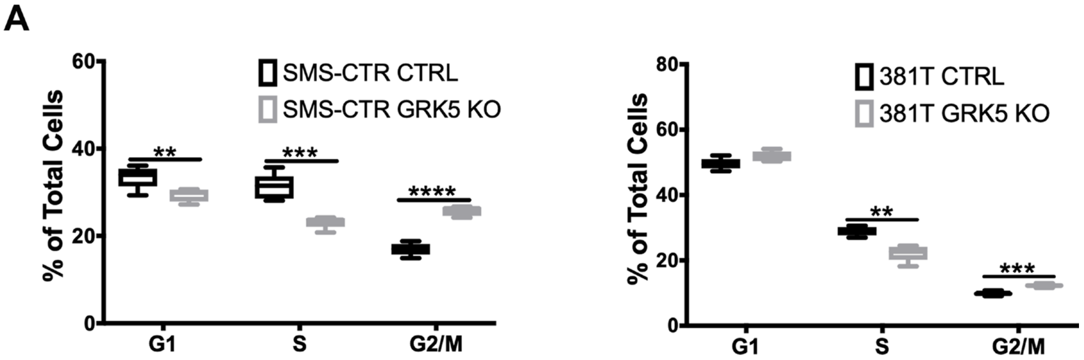

Supplementary Figure 2: Altered cell cycle progression day 6 following CRISPR/Cas9-mediated GRK5 targeting. (**A**) EdU flow cytometry-based cell cycle analysis of SMS-CTR and 381T cells with Cas9 only control (CTRL) or GRK5 knockout (GRK5 KO). Data shown are from 5 independent experiments. Two-tailed *t*-test, ** = $p < 0.01$, *** = $p < 0.001$, **** = $p < 0.0001$.

Supplementary Table 1: Results from siRNA kinome screen in ERMS cancer cell lines. see Supplementary Table 1
