# Supplementary material for: Characterization of GRK5 as a novel regulator of rhabdomyosarcoma tumor cell growth and self-renewal
Source: Oncotarget. 2020 Apr 21;11(16):1448–61. doi: 10.18632/oncotarget.27562 (PMC7185065; doi:10.18632/oncotarget.27562)
Supplement: Supplementary file 2 [file oncotarget-11-1448-s002.pdf]

Supplemental Table 1. Results from siRNA kinome screen in ERMS cancer cell lines.

Gene Symbol	Well	381T Sphere/Adherent	RD Sphere/Adherent	ABS delta
**AAK1**	O21	0.971441222	1.014572611	4.251187981
**AATK**	B17	0.984972181	1.706107654	42.26787629
**ABCC1**	F12	1.09789157	1.078228882	1.823609861
**ABL1**	F22	0.909051872	0.94742183	4.049933912
**ABL2**	H22	0.939642424	1.283201192	26.77356991
**ACVR1**	M10	1.060572377	1.324248314	19.91136659
**ACVR1B**	F09	0.947253898	1.111214261	14.7550629
**ACVR1C**	B20	0.95711407	0.971201455	1.450511056
**ACVR2A**	A16	1.136773721	0.721177522	57.62744754
**ACVR2B**	P04	0.89744617	1.001196068	10.36259549
**ACVRL1**	G03	1.011768864	1.028160901	1.594306618
**ADCK1**	J05	1.011809945	1.650469334	38.69562286
**ADCK2**	J16	0.846721051	0.864017162	2.001824947
**ADCK4**	B11	1.433446522	0.819391178	74.94043861
**ADCK5**	N18	1.164946378	1.229299857	5.234970003
**ADK**	G11	0.824273533	1.078883708	23.59940864
**ADRBK1**	J06	1.022138113	1.161876353	12.02694589
**ADRBK2**	N19	1.067124164	1.476942855	27.74776894
**AK1**	G06	0.966632458	0.896041947	7.878036464
**AK2**	O17	1.412798931	1.169500957	20.80357201
**AK3**	K03	0.708068838	1.270517786	44.26926994
**AK3L1**	F20	0.897852889	1.293842748	30.60571772
**AK3L2**	O17	1.036824823	1.551625298	33.17814398
**AK5**	M16	0.844438218	0.665702779	26.84913515
**AK7**	I16	1.029353641	1.283269533	19.78663763
**AKAP13**	I11	0.87211119	0.609041663	43.19401166
**AKT1**	P19	0.971537416	0.671582219	44.66395748

**AKT2**	C16	1.016055534	1.273716036	20.22903813
**AKT3**	F06	1.143406588	0.627626902	82.17934638
**ALDH18A1**	C10	0.907954259	0.854517643	6.253424533
**ALK**	H09	1.255026442	1.129392056	11.124072
**ALPK1**	D11	0.850863792	0.902277846	5.698250758
**ALPK2**	J14	1.161094312	1.041191251	11.51594972
**ALPK3**	I09	0.690584998	2.266286231	69.52790039
**ALS2CR2**	P03	1.550298012	0.805306552	92.51029406
**ALS2CR7**	D18	1.273367909	1.301536695	2.164271352
**AMHR2**	B07	1.173800667	0.831140105	41.22777376
**ANKK1**	G05	1.012571207	0.878682584	15.23742757
**ARAF**	E16	0.886262685	0.795893326	11.35445618
**ATM**	I03	1.248437698	0.937901944	33.1096183
**ATR**	B06	0.973461914	0.846907692	14.94309522
**AURKA**	O05	1.748137602	1.371398249	27.47118518
**AURKB**	B09	0.988184094	1.393637907	29.09319639
**AURKC**	L04	1.069852637	1.065219693	0.434928478
**AXL**	G16	0.655452599	0.730471974	10.26998676
**BAIAP2**	E22	1.314747709	1.082767157	21.42478654
**BCKDK**	I15	0.908984173	0.801919979	13.35098224
**BCR**	J09	1.008955825	0.832701119	21.16662291
**BLK**	G13	0.877030337	0.812078755	7.99818759
**BMP2K**	I17	0.890880965	1.347017843	33.86271981
**BMPR1A**	L09	0.948101554	0.791038189	19.85534543
**BMPR1B**	O10	1.465225406	1.019458488	43.72585282
**BMPR2**	A12	1.149691221	1.091825722	5.299884169
**BMX**	D20	0.917047448	0.758448336	20.91099731
**BRAF**	F10	1.095686971	1.05543774	3.813510677
**BRD2**	J19	0.844746267	0.6785324	24.49608404
**BRD3**	C15	0.924880793	1.458109147	36.56985179
**BRD4**	F16	0.915677955	1.11796661	18.09433779
**BRDT**	K17	0.979788267	1.014521836	3.423639446

**BRSK1**	P13	1.453420943	0.985389015	47.49717323
**BRSK2**	J05	0.942703932	0.976182256	3.42951569
**BTK**	K03	1.319805467	1.073726111	22.91826132
**BUB1**	N09	0.855649686	0.570301967	50.03449675
**BUB1B**	C12	1.324520899	1.088687893	21.66213178
**C1orf57**	C16	1.124901011	1.275292192	11.79268424
**C6orf199**	M08	0.718270771	0.952807392	24.61532333
**C9orf96**	H18	0.706186999	1.1774674	40.02492136
**CABC1**	H05	1.369493338	0.874127418	56.66976121
**CAMK1**	M22	1.162434417	1.262025917	7.891398972
**CAMK1D**	H22	1.010305024	1.082731653	6.689250127
**CAMK1G**	N05	1.029986404	1.284709007	19.82726061
**CAMK2A**	N22	0.939287127	1.222434898	23.16260532
**CAMK2B**	E12	1.325895275	0.817281432	62.23239924
**CAMK2D**	G12	0.911126586	0.866527465	5.146879111
**CAMK2G**	P22	0.928073637	1.458871794	36.3841538
**CAMK4**	I16	0.905775619	0.993127787	8.795662507
**CAMKK1**	J13	1.170993989	1.328998375	11.88898264
**CAMKK2**	O09	1.122960716	1.052435748	6.701118605
**CAMKV**	K09	0.63150568	1.526544838	58.63169792
**CARD14**	I14	1.013295141	0.957788563	5.795285187
**CARKL**	G17	1.102397178	1.292912424	14.73535585
**CASK**	H08	0.86042273	1.097407671	21.59497764
**CCRK**	G15	0.947366579	1.354904518	30.0787202
**CCT2**	A08	0.641821693	1.223696822	47.55059579
**CDC14B**	D17	0.852063163	0.734724725	15.97039464
**CDC2**	K16	0.992157158	1.181905298	16.05442838
**CDC2L1**	C04	0.744086683	1.080529578	31.13685192
**CDC2L2**	D14	0.786187054	1.40575618	44.07372597
**CDC2L2**	H16	0.681999092	1.39829788	51.22648029
**CDC2L5**	J03	1.506743557	0.758363858	98.68346063
**CDC2L6**	I08	0.697901021	1.285041933	45.69040878

**CDC42BPA**	E22	1.10858571	0.936107035	18.42510182
**CDC42BPB**	D10	0.840505728	1.457203911	42.3206511
**CDC42BPG**	I22	1.106802445	1.452349079	23.79225763
**CDC7**	M20	1.103054175	0.81911257	34.66454005
**CDK10**	D03	0.926572192	0.970722708	4.548210896
**CDK2**	G18	1.272423219	0.998880292	27.38495581
**CDK3**	I12	0.832243615	0.853514832	2.492190702
**CDK4**	G21	0.853883199	0.691793851	23.43029622
**CDK5**	D17	0.957110929	0.527381008	81.48376864
**CDK6**	K12	0.718708449	0.819926947	12.34481899
**CDK7**	M16	0.975133113	1.273368406	23.42097481
**CDK8**	D06	1.036075192	0.924328233	12.08953215
**CDK9**	M21	0.795952564	0.774035292	2.831559726
**CDKL1**	L07	1.094785919	0.926215754	18.19988103
**CDKL2**	H05	0.849762706	1.485135756	42.78215289
**CDKL3**	I20	1.052453288	1.254528436	16.1076579
**CDKL4**	I08	1.09481598	0.891011386	22.87339945
**CDKL5**	O19	1.102110034	1.096768388	0.487035032
**CERK**	E14	0.987087009	0.933863841	5.699242899
**CERKL**	O16	1.586105926	2.010129609	21.09434541
**CHEK1**	M12	1.09532239	1.073352969	2.046803035
**CHEK2**	E08	0.844973494	1.019523596	17.12075149
**CHKA**	A05	0.85221904	1.122507132	24.07896439
**CHKB**	M18	1.136840684	0.99189314	14.61322174
**CHUK**	F06	1.625765038	0.856293573	89.86070776
**CIT**	I13	0.909980806	1.4195849	35.89810614
**CKB**	O16	1.213451812	1.152855399	5.256202368
**CKM**	A18	1.476182157	0.885950594	66.62127295
**CKMT1B**	K05	0.533298526	1.524381979	65.01542704
**CKMT2**	I13	0.822952072	1.196015215	31.19217362
**CLK1**	D07	1.193968883	0.417021036	186.3090303
**CLK2**	P05	1.084120827	1.153006642	5.974450836

**CLK3**	N05	1.413491414	0.845533166	67.17161083
**CLK4**	F07	1.315535924	1.108767179	18.64852691
**CMPK**	O10	1.136157831	1.126058866	0.89684166
**COASY**	A16	0.974145267	1.44361578	32.52046143
**CRKL**	D21	1.115273852	1.325709177	15.87341541
**CRKRS**	E08	1.120920102	2.122005167	47.17637263
**CSF1R**	F21	1.204122704	1.090255981	10.44403563
**CSK**	H10	0.846242174	1.035637629	18.28781127
**CSNK1A1**	C18	1.062768851	1.435496725	25.96508005
**CSNK1A1L**	N18	0.983356439	1.089233543	9.720330816
**CSNK1D**	E18	1.249674033	0.998062802	25.20995982
**CSNK1E**	F18	0.775006532	0.942595966	17.77956192
**CSNK1G1**	K08	1.010153707	0.69482131	45.38323635
**CSNK1G2**	A14	1.126237755	1.745344502	35.47189375
**CSNK1G3**	P09	1.47539182	1.305905249	12.9784739
**CSNK2A1**	G18	0.686322216	0.778695183	11.86253227
**CSNK2A2**	I18	0.874871788	1.069257593	18.1795113
**DAK**	K10	1.286129972	1.097591716	17.17744892
**DAPK1**	F17	0.612901511	0.981079513	37.52784533
**DAPK2**	N22	1.192380261	1.473010965	19.05150136
**DAPK3**	C13	1.05865418	1.470491693	28.0067895
**DCAMKL1**	F15	0.768980803	0.726001105	5.920059568
**DCAMKL2**	K06	0.654103242	1.07784554	39.31382395
**DCAMKL3**	O18	0.859709218	1.027677211	16.34443107
**DCK**	K06	1.161344488	0.903745661	28.50346483
**DDR1**	L06	1.216227331	0.67171209	81.0637845
**DDR2**	J10	0.839511853	1.355641115	38.07270642
**DGKA**	C14	0.948563231	0.483581629	96.15369433
**DGKB**	H07	0.848170467	0.937727068	9.550391016
**DGKD**	B22	1.18981426	1.162158511	2.379688171
**DGKE**	K22	1.123496307	0.958034518	17.27096313
**DGKG**	E14	1.235581384	0.84241364	46.67157859

**DGKH**	D22	0.917448442	1.235630981	25.75061193
**DGKI**	N10	1.114425414	0.885582561	25.84093943
**DGKQ**	G14	0.809055211	0.795961303	1.64504331
**DGKZ**	I07	1.14006357	1.551444707	26.51600376
**DGUOK**	K18	1.259776643	0.920437051	36.86722438
**DLG1**	C10	0.866843546	0.777981112	11.42218401
**DLG2**	I14	0.947887748	1.347344103	29.64768642
**DLG3**	O12	1.004783435	1.107033155	9.236373731
**DLG4**	K08	1.069229064	0.948007078	12.78703384
**DLG5**	H15	2.911408112	1.112176827	161.7756495
**DMPK**	O13	0.845364132	1.30849991	35.39440654
**DTYMK**	I15	0.798644954	0.994141907	19.66489405
**DYRK1A**	L16	1.095184471	1.523086004	28.09437756
**DYRK1B**	D15	1.517756513	1.01844845	49.0263463
**DYRK2**	M09	1.148294998	1.326576227	13.43919976
**DYRK3**	C22	1.066168443	1.582587231	32.63130002
**DYRK4**	P03	0.940549511	0.886013815	6.155174415
**EEF2K**	E08	0.767043001	0.998334052	23.16770136
**EGFR**	H21	1.077368567	0.693471252	55.35879304
**EIF2AK1**	A21	1.00533174	0.920673716	9.195225412
**EIF2AK2**	G17	1.090740538	1.148842666	5.057448646
**EIF2AK3**	P15	1.388238193	1.351163405	2.74391588
**EIF2AK4**	A10	1.146727389	1.221259562	6.102893757
**EPHA1**	J21	1.526429476	0.70353411	116.9659516
**EPHA10**	C08	1.013174262	1.316189569	23.02216293
**EPHA2**	M04	0.863443511	1.452095382	40.53809951
**EPHA3**	N12	1.31611957	1.044608946	25.99160435
**EPHA4**	B11	1.492872351	1.539522153	3.03014809
**EPHA5**	D11	1.040039858	1.091659342	4.728534115
**EPHA6**	K03	1.353959711	0.884875774	53.01127573
**EPHA7**	F11	0.883017908	0.65212847	35.40551408
**EPHA8**	P05	1.32499334	1.733096799	23.54764369

**EPHB1**	H11	0.971491371	1.211071457	19.78248969
**EPHB2**	C22	0.852953995	0.767662247	11.11058266
**EPHB3**	A15	1.047806899	0.888721403	17.90049122
**EPHB4**	J11	1.23823741	1.776991942	30.31834413
**EPHB6**	L11	1.279242592	0.968064569	32.1443459
**ERBB2**	N11	1.123286871	1.03691899	8.329279458
**ERBB3**	N06	1.039870023	0.861067364	20.76523475
**ERBB4**	P12	1.132426532	1.111490159	1.883630969
**ERN1.00**	K14	0.659103289	0.99222489	33.57319533
**ERN2.00**	B17	1.05497013	0.729315996	44.6519939
**ETNK1**	K12	0.955019519	1.186931816	19.53880532
**ETNK2**	E12	1.080820165	1.168557277	7.50815675
**EVI5L**	P18	1.108951086	1.249961723	11.28119642
**FASTK**	E11	0.810577511	1.023210693	20.78097734
**FER**	L21	0.992685059	0.853702299	16.28000293
**FES**	M18	1.605251751	0.442066233	263.1247152
**FGFR1**	I06	1.005962179	1.047878315	4.000095682
**FGFR2**	J21	1.141476956	1.284060721	11.10412947
**FGFR3**	L21	1.143731281	1.331727085	14.11669142
**FGFR4**	A09	0.848122514	1.110428899	23.62207835
**FGR**	N21	1.341058418	0.906638069	47.91552039
**FLJ10986**	G12	0.988486085	1.671680907	40.86873392
**FLJ23356**	M10	0.787152719	1.652882904	52.37698222
**FLJ25006**	B18	0.862536456	0.81012995	6.468901224
**FLJ30698**	M17	1.275382301	0.795214638	60.38214589
**FLJ32786**	E16	1.158148739	1.082191942	7.018791627
**FLJ40852**	L18	1.111753972	1.273808441	12.72204388
**FLT1**	M03	0.998605755	0.982142352	1.676274622
**FLT3**	D10	0.955594542	1.34774886	29.09698755
**FLT4**	M05	1.054961827	0.862165362	22.36188947
**FN3K**	D09	0.987639011	0.915208586	7.914089378
**FRAP1**	H17	0.720684875	0.92714274	22.26818554

**FRK**	O03	1.181269835	0.718971304	64.29999769
**FUK**	G16	0.900788016	0.754335257	19.41481038
**FYN**	A05	1.166964529	1.267116883	7.903955441
**GAK**	P21	1.196307776	0.980956419	21.95320327
**GALK1**	I21	0.916438979	1.220549701	24.91588194
**GALK2**	A08	0.834746703	1.294481473	35.51497488
**GCK**	K21	1.335932423	0.862747611	54.84626152
**GK**	M21	0.960216229	0.865204221	10.98145448
**GK2**	P15	2.070952575	1.365174198	51.69877789
**GNE**	J06	1.079030505	0.991437005	8.835004129
**GPR125**	D20	1.221125394	1.002596895	21.79624741
**GRK1**	C19	1.187339022	1.224818298	3.059986636
**GRK4**	G17	1.166874608	1.196336793	2.462699892
**GRK5**	D04	0.706363289	0.608153712	16.14880837
**GRK6**	C05	1.112027992	1.182380354	5.950061847
**GRK7**	P16	1.031682308	1.285317738	19.73328639
**GSG2**	O19	0.919864312	1.447037335	36.43119704
**GSK3A**	F05	1.322798485	1.304436916	1.407624149
**GSK3B**	E05	1.381284331	0.809706604	70.5907206
**GUCY2C**	J17	0.866996176	0.789361694	9.835096219
**GUCY2D**	K21	1.031950932	1.24241326	16.93980058
**GUCY2F**	O14	1.104473636	1.516317985	27.16081672
**GUK1**	K09	1.115160362	1.364325705	18.26289293
**HCK**	G05	1.043436126	0.774969347	34.64224488
**HERC2**	P13	1.294613083	1.463268324	11.52592713
**HIPK1**	B20	0.898333527	1.083688315	17.10406817
**HIPK2**	J09	1.019138742	1.450316039	29.72988548
**HIPK3**	B08	1.004969206	1.183809354	15.10717481
**HIPK4**	F18	0.798805522	0.851409639	6.178473219
**HK1**	F17	0.668124367	0.818763074	18.39832685
**HK2**	O21	0.771196842	0.972079873	20.66528033
**HK3**	H17	1.507392792	1.649288399	8.603444178

**HKDC1**	O05	0.898981811	1.482079095	39.34319609
**HSPB8**	I19	1.361014816	1.214806532	12.03552011
**HUNK**	I21	1.183584417	1.33917366	11.61830225
**ICK**	C10	0.788703611	1.136938489	30.62917485
**IGF1R**	M06	1.109842138	0.89243445	24.36119401
**IHPK1**	G08	1.264229325	0.426830304	196.190152
**IHPK2**	M10	1.215345486	1.22199313	0.544000157
**IHPK3**	B16	0.846797147	1.04728518	19.14359498
**IKBKB**	O21	1.106962558	0.928844246	19.17633795
**IKBKE**	A19	1.015388996	1.196965941	15.16976707
**ILK**	P11	1.055443168	1.068033815	1.178862244
**INSR**	C04	1.254798985	1.27187142	1.342308241
**INSRR**	G08	0.802561126	0.789221038	1.690285329
**IPMK**	A07	0.807322016	1.131655384	28.66008265
**IRAK1**	H06	1.056844578	1.380902953	23.46713609
**IRAK2**	E13	0.557459725	0.952487708	41.47328931
**IRAK3**	O13	1.182017487	1.329662642	11.10395602
**IRAK4**	I06	1.445198929	1.037841263	39.25047896
**ITK**	L06	1.060770695	1.17381918	9.630826176
**ITPK1**	E05	0.745599396	1.22379596	39.07486049
**ITPKA**	M08	1.197509316	1.684017008	28.88971368
**ITPKB**	A10	1.52623036	1.145738261	33.20933865
**ITPKC**	O14	1.281752796	1.0740393	19.33946886
**JAK1**	C21	0.713802607	0.866553731	17.62742674
**JAK2**	L17	1.445132851	0.984099689	46.84821747
**JAK3**	E04	1.071379259	0.828349587	29.33902249
**KALRN**	F05	0.971392559	0.827193652	17.43230337
**KDR**	J17	1.157411341	1.189856808	2.726837959
**KHK**	N21	1.058877159	1.454042118	27.17699535
**KIAA0999**	F11	1.22894545	0.728322843	68.73635929
**KIAA1639**	C03	0.9447664	1.366880147	30.88154787
**KIAA1804**	B15	1.035530047	1.051362621	1.505909918

**KIAA2002**	L09	1.17481995	1.271062425	7.571813444
**KIT**	P21	1.075645244	0.52532837	104.75674
**KSR1**	E19	1.245342595	1.222318801	1.883616108
**KSR2**	I03	1.173495033	1.036723671	13.1926536
**LATS1**	G15	0.903794094	1.260166054	28.27976192
**LATS2**	G21	1.09787013	1.350784261	18.72350298
**LCK**	F04	1.204549064	1.14841362	4.888085856
**LIMK1**	I05	1.210993539	1.099194877	10.17095918
**LIMK2**	N06	1.327993491	1.048342775	26.67550377
**LMTK2**	C04	0.99076297	0.923743418	7.255212991
**LMTK3**	A20	1.301675987	1.131271393	15.0631046
**LOC122134**	A18	1.094170032	0.975006306	12.22184159
**LOC126520**	C09	1.024184186	0.830726908	23.28771058
**LOC390975**	C07	0.607086638	1.143172664	46.89458055
**LOC400301**	E09	0.783494831	1.065568769	26.47167844
**LOC442075**	E03	0.983204758	0.830906865	18.32911716
**LOC91461**	J16	0.880931173	0.885213021	0.483708161
**LRRK1**	M09	1.084237292	1.489146516	27.19069072
**LRRK2**	C07	0.97229808	0.925200882	5.090483491
**LTK**	K05	1.331161364	0.417022835	219.2058692
**LYK5**	J04	1.219809892	0.815265967	49.62109806
**LYN**	M05	1.088534808	0.518568933	109.9113039
**MAGI1**	E10	0.928136168	1.444969222	35.7677552
**MAGI2**	G10	0.911236357	0.811376032	12.30752716
**MAK**	N08	0.964216436	0.81542956	18.24644141
**MAP2K1**	D21	0.765192476	0.787064047	2.778880739
**MAP2K2**	J11	1.117635161	2.095983608	46.67729476
**MAP2K3**	J18	0.910731604	0.759199281	19.9594924
**MAP2K4**	G19	1.087858043	0.832466522	30.67889395
**MAP2K5**	G20	0.928147422	1.134092178	18.15943711
**MAP2K6**	E17	0.964873616	0.704065727	37.04311664
**MAP2K7**	L18	0.85549429	0.989327983	13.52773762

**MAP3K1**	C20	1.103692916	0.988386847	11.66608689
**MAP3K10**	E07	1.037223084	0.794631941	30.52874294
**MAP3K11**	L17	1.218455207	0.914743385	33.20186043
**MAP3K12**	P12	1.076360882	0.833830275	29.08632767
**MAP3K13**	P10	1.230995833	1.348677368	8.725699582
**MAP3K14**	N08	1.130591961	0.975541151	15.89382567
**MAP3K15**	H04	1.025866323	1.279469693	19.82097516
**MAP3K2**	N20	0.933704339	1.176328476	20.62554313
**MAP3K3**	C07	1.015925518	1.395109093	27.17949275
**MAP3K4**	B10	0.811775662	0.920999821	11.85930291
**MAP3K5**	J14	1.083219705	1.41666077	23.53711428
**MAP3K6**	B15	1.080579912	0.94912097	13.85059926
**MAP3K7**	D18	1.135095447	1.026922321	10.53372047
**MAP3K8**	B21	0.910914294	0.951587058	4.274203187
**MAP3K9**	N15	0.897472893	1.313046092	31.64955152
**MAP4K1**	K13	0.814424623	1.095912212	25.68523158
**MAP4K2**	F13	1.014217272	0.715318727	41.78536565
**MAP4K3**	G22	1.166183537	1.0853122	7.451435305
**MAP4K4**	H20	0.819242137	0.704338777	16.31364954
**MAP4K5**	E07	0.985563815	1.106823298	10.9556316
**MAPK1**	G15	0.968381259	0.818690089	18.28422896
**MAPK10**	C17	1.913633349	1.958474025	2.289572158
**MAPK11**	O15	1.301151771	1.258630891	3.37834396
**MAPK12**	E19	1.240716284	1.155346064	7.389147019
**MAPK13**	B21	0.834459618	0.810569098	2.947376045
**MAPK14**	O12	1.009331904	1.030137085	2.019651681
**MAPK15**	P16	1.067364383	0.988157854	8.015574467
**MAPK3**	I15	1.019402921	0.778294504	30.97907221
**MAPK4**	K15	0.840409637	0.887592259	5.315799253
**MAPK6**	E20	1.154498077	1.155532863	0.089550534
**MAPK7**	B18	0.925811992	0.774594674	19.52212223
**MAPK8**	M15	0.947615496	0.80541545	17.65549026

**MAPK9**	A17	1.019463641	0.934879998	9.047540137
**MAPKAPK2**	G11	0.631579281	1.143849919	44.78477721
**MAPKAPK3**	L13	0.969166229	0.691166066	40.22190568
**MAPKAPK5**	O22	1.328537546	1.100738797	20.69507767
**MARK1**	B05	0.944313919	0.841371437	12.23508151
**MARK2**	G22	1.446730999	1.750803413	17.36759318
**MARK3**	O05	1.02317258	0.862665929	18.60588744
**MARK4**	N11	1.791269862	1.691159497	5.919628867
**MARVELD3**	E10	0.995294724	1.157274157	13.99663444
**MAST1**	E04	1.421282125	1.178381336	20.61308864
**MAST2**	K04	1.241639914	1.405396723	11.65199877
**MAST3**	E07	0.843530895	1.324185544	36.29813446
**MAST4**	G07	0.629370553	0.973833789	35.37187142
**MASTL**	H15	0.889426422	1.629507729	45.41747756
**MATK**	A07	0.893665919	1.15427731	22.57788392
**MELK**	A19	0.999742672	1.500138307	33.35663335
**MERTK**	C17	1.171946231	1.202702122	2.55723259
**MET**	G04	1.320077895	1.057285492	24.8553872
**MGC16169**	E11	0.748565395	1.05639131	29.13938347
**MGC26597**	K16	0.980671071	1.330205274	26.27671154
**MGC42105**	F22	1.240647691	1.140204268	8.809248127
**MINK1**	A06	1.095338426	1.255772185	12.77570575
**MIP**	E15	1.386404737	1.600331216	13.3676377
**MKNK1**	H03	1.118878046	0.8392634	33.31667344
**MKNK2**	O06	0.661512232	0.88979957	25.65604051
**MLCK**	K05	1.283202419	1.043866409	22.92783902
**MLKL**	N20	1.169389543	1.279477809	8.604155915
**MOS**	C06	0.706782523	0.963703426	26.65974783
**MPP1**	N17	1.02349024	1.51046011	32.2398365
**MPP2**	H04	1.051675463	0.829882274	26.72586168
**MPP3**	K13	1.252006384	1.622365192	22.82832558
**MPP4**	C11	0.786863102	1.06447276	26.07954553

**MPP5**	H09	1.149113198	1.277361101	10.04006644
**MPP6**	A08	1.073042329	1.041120621	3.066091188
**MST1R**	G07	1.27906194	0.920406638	38.96704858
**MULK**	I05	0.669366261	1.911872756	64.98897435
**MUSK**	E06	0.669491369	1.076142635	37.78785948
**MVK**	E06	0.833110812	0.711660752	17.06572404
**MYLK**	P14	1.001742747	1.070210877	6.397629831
**MYLK2**	L15	1.035522573	1.148981679	9.874753273
**MYO3A**	A22	1.228398525	1.038105667	18.33077923
**MYO3B**	N16	0.976936369	1.048026792	6.783263909
**NAALADL1**	H06	0.73994378	0.606849074	21.93209344
**NADK**	G14	0.9845625	1.215773744	19.01762107
**NAGK**	G05	0.714441259	1.291731964	44.69121464
**NDUFA10**	B13	1.060844199	1.226340324	13.49512211
**NEK1**	K15	0.603371463	0.978711504	38.35042702
**NEK10**	J20	0.893240402	0.716794211	24.61601783
**NEK11**	P09	1.230513638	1.255439737	1.98544764
**NEK2**	I07	0.899943525	0.910794738	1.191400442
**NEK3**	K07	1.144763393	1.356697589	15.62132921
**NEK4**	M19	1.324676825	0.995501862	33.0662328
**NEK5**	G07	1.919318911	1.20053732	59.8716574
**NEK6**	O19	1.126283768	1.232551174	8.621743961
**NEK7**	N16	0.868083539	1.164930377	25.48193811
**NEK8**	E17	0.72818212	0.924960085	21.27421152
**NEK9**	J15	1.641854857	2.100933262	21.85116555
**NLK**	O06	0.996314637	1.185712697	15.97335178
**NME1**	I04	0.984648687	1.308275282	24.73688835
**NME1-NME2**	I10	0.969525775	0.978422767	0.909319799
**NME3**	M07	2.045574047	0.78855248	159.4087393
**NME4**	H12	1.444593884	1.10659592	30.54393733
**NME5**	O20	1.340649087	1.719520279	22.03354017
**NME6**	F08	0.824523315	0.606591209	35.92734339

**NME7**	I17	0.959250271	1.08726759	11.77422376
**NPR1.00**	O06	1.027877317	1.092159056	5.885748848
**NPR2.00**	B07	1.45637129	1.178246254	23.60500063
**NRBP1**	M17	0.816477292	1.080007533	24.40077809
**NRBP2**	I05	1.38970997	1.110382355	25.15598458
**NRK**	A07	1.099445097	1.040932789	5.621141865
**NTRK1**	A09	1.001147951	0.998863626	0.228692364
**NTRK2**	H10	0.553718904	0.914197365	39.431142
**NTRK3**	C09	0.837991603	1.075444079	22.07948144
**NUAK1**	E03	0.836901126	1.238590216	32.43115313
**NUAK2**	O09	0.778669474	1.051919188	25.97630278
**OXSM**	M07	0.745613286	1.406100273	46.97296486
**OXSR1**	L12	0.989491067	1.09403041	9.555432962
**PAK1**	E09	0.876873714	0.889416531	1.410229825
**PAK2**	P17	0.989267216	1.283212172	22.90696443
**PAK3**	B19	0.92285794	1.198895472	23.02432013
**PAK4**	L08	1.145469423	0.832211764	37.64158035
**PAK6**	C09	0.715069678	1.194416304	40.13229093
**PAK7**	E09	0.755665734	1.234836128	38.80437115
**PAN3**	O03	0.952721475	1.30745492	27.13160048
**PANK1**	I18	1.075014502	0.999560116	7.548759148
**PANK3**	K14	0.994609182	0.926639404	7.335083877
**PAPSS1**	D14	0.939033035	1.006037167	6.660204479
**PAPSS2**	N04	1.036978387	0.825299614	25.64871835
**PASK**	G03	0.576519177	1.672016773	65.51953386
**PBK**	N03	1.143938047	1.248469691	8.37278185
**PCK1**	G09	0.673869435	0.505532323	33.29898082
**PCK2**	J10	0.788019176	1.324323247	40.49646283
**PCTK1**	O10	1.155749871	0.998418835	15.75801967
**PCTK2**	I09	0.824414466	1.255545528	34.33814644
**PCTK3**	M07	1.229028629	0.95896553	28.16191925
**PDGFRA**	L10	1.051620084	0.851645606	23.48094992

**PDGFRB**	K09	1.283316311	0.831834547	54.2754285
**PDIK1L**	P20	1.304172157	1.219411712	6.9509292
**PDK1**	E21	0.913036987	1.45620497	37.30024239
**PDK2**	M09	1.073881259	1.057980714	1.502914482
**PDK3**	J04	1.046755328	0.885573953	18.20078084
**PDK4**	O09	1.096299318	1.157578384	5.293729335
**PDPK1**	A11	0.938674003	0.760392824	23.44593126
**PDXK**	F03	0.979375976	0.883357685	10.86969563
**PDZD2**	E05	0.985852901	1.096700368	10.10736118
**PFKFB1**	C11	1.214996851	0.80112074	51.66213908
**PFKFB2**	O03	0.63447125	1.272634866	50.14506776
**PFKFB3**	L10	1.151439228	1.086777933	5.949816723
**PFKFB4**	C05	0.585192932	1.441539438	59.4050002
**PFKL**	D19	0.830554107	1.145330554	27.48345845
**PFKM**	K04	1.614514985	1.174791636	37.42990119
**PFKP**	F19	1.052662764	1.08426869	2.91495329
**PFTK1**	O15	0.978015987	1.443535132	32.24854975
**PGK1.00**	M04	1.041423224	0.966148081	7.791263558
**PGK2.00**	L16	1.000383391	1.076589722	7.078493225
**PHKA1**	E11	0.929284032	1.129216094	17.70538542
**PHKA2**	O04	0.94959418	1.188988632	20.13429276
**PHKB**	H20	0.968737627	1.119348026	13.4551896
**PHKG1**	M15	1.433910627	1.926718519	25.57757592
**PHKG2**	A06	0.948652754	0.965578091	1.75287084
**PI4K2B**	F03	1.02234182	0.990011659	3.265634424
**PI4KII**	L03	1.268267565	1.422010057	10.81163188
**PIK3C2A**	G11	1.004498521	0.679958875	47.7293051
**PIK3C2B**	I11	0.869241789	0.496860681	74.94678555
**PIK3C2G**	D13	0.837155292	1.286670388	34.93630542
**PIK3C3**	K11	0.763848736	0.648186946	17.84389365
**PIK3CA**	N10	1.002601611	1.043471249	3.916699932
**PIK3CB**	P10	1.075475098	1.072590761	0.268913074

**PIK3CD**	P17	1.255770039	1.27980986	1.878390041
**PIK3CG**	O11	1.049031667	0.979056755	7.147176311
**PIK3R2**	A06	0.823984019	1.470280451	43.95735737
**PIK3R3**	I22	1.14384112	0.835819353	36.85267234
**PIK3R4**	K21	1.1743142	1.074213778	9.318482421
**PIK4CA**	D16	1.019429192	1.229271415	17.0704549
**PIK4CB**	A13	1.202573678	0.987499146	21.77971833
**PIM1**	M11	1.220582952	1.050846206	16.15238704
**PIM2**	A13	0.795557257	1.250235665	36.36741622
**PIM3**	C18	1.62822501	1.735952035	6.205645286
**PIN1**	P14	1.035434154	1.312825399	21.12933264
**PINK1**	L13	1.240157655	1.364424543	9.107640936
**PIP5K1A**	K11	1.056955877	1.510649159	30.03300135
**PIP5K1B**	A22	1.574282021	0.749691461	109.9906565
**PIP5K1C**	A17	1.107353664	1.35773751	18.44125571
**PIP5K2A**	B19	0.980587186	0.839305178	16.83321064
**PIP5K2B**	D08	1.007384919	1.377801756	26.88462507
**PIP5K2C**	N09	1.100447294	0.808656432	36.08341579
**PIP5K3**	J20	1.474520425	1.246330748	18.30891819
**PKD2L1**	G06	0.996879617	0.549269101	81.49202543
**PKD2L2**	M19	1.233712271	1.128677316	9.306021636
**PKLR**	C06	0.9233442	0.873271693	5.733897915
**PKM2**	C13	1.157407315	1.057916887	9.404370907
**PKMYT1**	N07	1.108569562	1.430883079	22.52549645
**PKN1**	E15	0.919242617	0.808895634	13.64168369
**PKN2**	F12	0.749481196	1.152076776	34.94520408
**PKN3**	K17	0.967861954	1.033821347	6.380153904
**PLK2**	C11	0.876462696	0.892523934	1.799530271
**PLK3**	F07	1.115867682	0.992270789	12.45596408
**PLK4**	G19	0.968905724	0.799318534	21.21647157
**PMVK**	A11	1.013915475	1.320891402	23.24005794
**PNCK**	K18	0.850167959	1.054885505	19.40661289

**POLK**	M06	0.982692499	0.958828668	2.488852523
**POLR2K**	F19	1.143422178	1.018708892	12.24228895
**POLR3K**	K08	0.795919325	1.146798587	30.59641562
**PRKAA1**	B16	1.158758523	1.415867667	18.15912254
**PRKAA2**	B12	1.025924084	0.944505537	8.620229767
**PRKAB2**	L04	1.224176237	0.983748272	24.43998856
**PRKACA**	H19	1.086599001	1.201770096	9.583454941
**PRKACB**	J19	1.021589651	0.909671315	12.30316201
**PRKACG**	E13	0.899551469	0.401270469	124.1758461
**PRKAG1**	G13	0.846369736	0.723029523	17.05880728
**PRKAG2**	K06	1.060558314	0.806791487	31.45383059
**PRKAG3**	K07	0.592099965	1.870263798	68.34136631
**PRKAR1A**	K07	1.393376353	1.135479096	22.7126381
**PRKAR1B**	I13	0.987740867	1.337587356	26.15503857
**PRKAR2A**	J07	1.346232681	1.218333309	10.49789667
**PRKAR2B**	K13	1.000164319	1.258430534	20.52288211
**PRKCA**	O13	1.152224658	1.344123894	14.27690087
**PRKCB1**	A15	0.939592546	1.230477477	23.64000451
**PRKCD**	O15	0.877461341	1.479307646	40.68432329
**PRKCE**	N04	1.083628966	0.919316625	17.87331332
**PRKCG**	C15	1.007980543	1.304080836	22.70567016
**PRKCH**	D12	0.977553705	1.319845067	25.93420776
**PRKCI**	G21	0.97686883	1.163333113	16.02845137
**PRKCQ**	H12	0.58546434	0.579290766	1.065712441
**PRKCZ**	P19	1.132711139	1.157998119	2.183680599
**PRKD1**	N19	1.363102986	1.083037308	25.85928266
**PRKD2**	C08	1.00708853	0.915131937	10.04845196
**PRKD3**	H08	0.985835923	0.844853009	16.68727126
**PRKDC**	C13	0.957228923	1.018077705	5.976830861
**PRKG1**	J12	0.911674738	1.2542858	27.31523081
**PRKG2**	L12	0.965772158	0.774984875	24.61819434
**PRKX**	H19	0.924964413	0.675337636	36.96325568

**PRKXP1**	G09	0.808774073	0.792690887	2.028935404
**PRKY**	A10	0.782676351	1.423836292	45.03045361
**PRPF4B**	B05	0.855791137	1.039946385	17.70814833
**PRPS1**	I17	0.938598028	0.80517436	16.57077947
**PRPS1L1**	A05	0.891264181	1.30363278	31.63226677
**PRPS2**	K17	1.137127623	0.676223674	68.15850536
**PSKH1**	K11	1.179608303	1.345706659	12.34283524
**PSKH2**	I11	0.793833304	1.188218644	33.19131052
**PTK2**	P06	0.922449763	1.103210058	16.38493894
**PTK2B**	B10	1.081916641	0.931615124	16.13343468
**PTK6**	L14	1.112653206	0.846602206	31.42573909
**PTK7**	M17	1.566696999	1.09203396	43.46595955
**PTK9**	M11	0.69744584	1.600317625	56.41828667
**PTK9L**	C08	0.749743592	1.161972222	35.47663377
**PXK**	D03	1.360888072	0.998882314	36.24108193
**RAB32**	M11	0.955765206	1.086675425	12.04685554
**RAB38**	F09	1.321234874	1.21706384	8.55920872
**RAD18**	G10	0.886253829	1.383925246	35.96085974
**RAF1**	O17	1.086037567	1.162336957	6.564309025
**RAGE**	C19	1.288900704	0.98333301	31.07469099
**RALB**	A19	1.200586688	1.165168804	3.039721313
**RAPGEF3**	F10	0.982175018	0.756131361	29.89476024
**RAPGEF4**	D22	1.0260444	1.111320705	7.673419902
**RBKS**	C14	1.106526148	1.244022655	11.05257259
**RET**	L07	0.855357413	1.147759172	25.47588086
**RFK**	I12	0.994444305	1.234474507	19.44391732
**RIOK1**	B13	1.252791373	1.203060769	4.133673425
**RIOK2**	E10	1.047624822	1.156467682	9.411664663
**RIOK3**	N03	1.30765551	1.19557123	9.374956298
**RIPK1**	I04	0.823084236	1.672827876	50.79683642
**RIPK2**	L03	1.317036723	0.513776234	156.3444229
**RIPK3**	B22	0.841362509	0.830963669	1.25141927

**RIPK4**	D07	1.079299907	0.537783256	100.6942194
**RIPK5**	E19	0.842874146	1.922664125	56.1611342
**RNASEL**	N07	0.971428965	1.062882828	8.604322161
**ROCK1**	P04	1.084976438	0.958769981	13.16337178
**ROCK2**	D12	0.90834012	0.878183117	3.434022147
**ROR1**	N17	1.002030849	1.524758015	34.28263113
**ROR2**	C15	1.055624799	1.360255572	22.39511301
**ROS1**	F21	1.070252655	0.674496353	58.67434274
**RP11-145H9.1**	M08	1.080761606	1.575514577	31.40262733
**RP6-213H19.1**	M20	1.038084167	1.513680539	31.41986434
**RPS6KA1**	I21	1.128257891	1.397750889	19.28047405
**RPS6KA2**	G08	0.724160433	0.781764427	7.368459245
**RPS6KA3**	H13	1.219761452	0.997744991	22.25182423
**RPS6KA4**	D05	1.008061059	0.98668358	2.166599167
**RPS6KA5**	J15	1.053339239	1.00455209	4.856607143
**RPS6KA6**	E21	1.122811495	1.341607942	16.30852357
**RPS6KB1**	A13	1.113033976	1.090209092	2.093624465
**RPS6KB2**	L08	0.830939478	1.022125655	18.70476262
**RPS6KC1**	C17	1.07057569	1.007880548	6.220493364
**RPS6KL1**	P11	1.795031704	1.493162524	20.21676645
**RYK**	O11	0.760953274	0.98021357	22.3686249
**SBK1**	E18	1.241813687	1.223527548	1.494542549
**SCYL1**	G09	0.536043803	1.17524427	54.38873292
**SCYL2**	A09	0.761833248	0.838657008	9.160331292
**SCYL3**	L05	0.94587421	1.153853527	18.02475902
**SGK**	F14	1.000904745	1.40583466	28.80352338
**SGK2**	L22	0.99794317	0.914089468	9.173467736
**SGK3**	E17	1.243719312	1.511650723	17.72442582
**SKP1A**	J22	0.873252394	0.999411827	12.62336801
**SLK**	C03	0.587010896	1.301391676	54.89360302
**SMG1**	I04	1.368773452	1.374223098	0.396561976
**SNF1LK**	C03	0.935496508	1.043629581	10.36125035

**SNF1LK2**	M04	1.512577981	1.133256982	33.47175491
**SNRK**	O22	1.10147063	1.527592387	27.89499092
**SPEG**	J08	1.401357438	1.05518373	32.80696043
**SPHK1**	A14	0.816948341	1.321543028	38.18223671
**SPHK2**	M12	1.255009303	1.220841353	2.798721553
**SRC**	B06	0.939955012	1.215001728	22.63755766
**SRMS**	H16	0.827344287	0.705259237	17.31066298
**SRPK1**	H21	0.847371808	1.274517628	33.5143124
**SRPK2**	P18	1.209238376	1.201621725	0.633864272
**STC1**	I19	1.031113235	1.165700184	11.54558875
**STK10**	K15	0.875072864	1.057528266	17.25300479
**STK11**	D04	0.983215013	0.983642704	0.043480316
**STK16**	J08	1.212255725	1.212323535	0.005593335
**STK17A**	L15	1.32896665	0.956542107	38.93446415
**STK17B**	D09	0.626254289	0.845776913	25.95514502
**STK19**	D15	1.04011278	1.192316853	12.76540479
**STK23**	P22	1.16483661	1.141300168	2.062248187
**STK24**	F08	1.077637349	1.179222624	8.614596848
**STK25**	B14	0.936977576	0.781277087	19.92897172
**STK3**	A17	1.147591499	1.378369819	16.74284483
**STK31**	L11	1.102120886	0.833298935	32.2599657
**STK32A**	H18	0.617892337	0.860966855	28.23273824
**STK32B**	H03	1.466683334	1.372629659	6.852079438
**STK32C**	G03	0.902211882	1.024653886	11.94959642
**STK33**	K10	0.65063384	1.470952796	55.76786409
**STK35**	I06	0.511167808	1.370697379	62.70746442
**STK36**	I03	0.811797494	1.797991494	54.84975896
**STK38**	A15	1.130481416	1.550037984	27.06750238
**STK38L**	G04	1.344219558	1.298471608	3.523215235
**STK39**	J22	1.093930397	1.276839453	14.32514132
**STK4**	G06	0.891070233	1.506097194	40.83580816
**STK40**	D13	0.750869446	0.655677086	14.51817714

**STYK1**	J03	1.496496757	1.293394426	15.70304669
**SYK**	A21	1.058272606	1.08768057	2.703731698
**TAF1**	E15	0.85305977	1.059734853	19.50252765
**TAF1L**	J18	1.039263531	1.103053531	5.783037511
**TAF9**	I08	1.159492418	1.059784003	9.408371432
**TANK**	K04	0.754384871	1.376153443	45.1816311
**TAOK1**	H07	1.336727248	1.555756256	14.07861977
**TAOK2**	N15	0.974130926	0.807735138	20.60029079
**TAOK3**	I19	1.33079713	1.790213764	25.66266907
**TBK1**	L22	1.718637155	1.697154852	1.265783372
**TCEB3C**	F20	1.048738353	0.998577925	5.023186179
**TEC**	C21	1.357664953	1.565709441	13.28755405
**TEK**	F04	1.307531056	1.086623052	20.32977342
**TESK1**	N12	0.976300332	0.911918338	7.060061353
**TESK2**	G13	1.00336017	1.031273509	2.70668636
**TEX14**	M19	1.055059643	1.080908996	2.391445852
**TGFBR1**	J13	0.78641972	1.237272849	36.43926478
**TGFBR2**	E21	1.068573734	0.889273926	20.16249459
**TIE1**	D06	1.041419552	1.376324218	24.33326843
**TJP1**	P06	1.019430263	1.209410948	15.7085303
**TJP2**	B12	0.943513693	1.099447625	14.18293408
**TJP3**	C21	1.257219901	1.356411357	7.312785741
**TK1**	E04	0.863913812	1.62374839	46.79509357
**TK2**	O04	0.463508467	1.326836958	65.06665989
**TLK1**	M15	1.023404724	1.102610275	7.183458455
**TLK2**	O11	1.358936224	1.371899426	0.94490906
**TNIK**	C19	1.204936983	1.446347571	16.69104946
**TNK1**	L05	1.336002985	0.76939626	73.64303082
**TNK2**	H14	0.65412592	0.7256753	9.859696192
**TNNI3K**	G19	1.265833753	1.078038751	17.42006051
**TP53RK**	F14	1.579719242	1.246002707	26.78297034
**TPK1**	M05	0.694201607	1.202944289	42.29145824

**TRIB1**	H11	1.31051584	1.67742404	21.87331237
**TRIB2**	B09	1.054509571	0.829900731	27.06454305
**TRIB3**	I10	0.705931613	2.134894068	66.93364683
**TRIM24**	C06	0.831390186	0.717562107	15.86316746
**TRIM28**	D08	1.047732872	0.839467811	24.80917779
**TRIM33**	E06	0.9887542	1.588133756	37.74112559
**TRIO**	E13	0.877180717	1.033186047	15.09944221
**TRPM6**	K22	0.97664277	1.746737918	44.08761843
**TRPM7**	M22	1.214722012	1.019773374	19.11685907
**TRRAP**	I20	1.010176807	0.9068753	11.39092734
**TSSK1**	F13	0.875915759	0.725817426	20.6799022
**TSSK2**	L14	0.983084928	0.85365319	15.16209845
**TSSK3**	H14	0.884425366	0.480215679	84.17252986
**TSSK4**	M03	1.148371425	0.94315814	21.75809933
**TSSK6**	H13	0.842083592	1.101832238	23.57424632
**TTBK1**	F15	0.878212894	0.995140808	11.74988637
**TTBK2**	E03	1.049668365	1.153028928	8.964264543
**TTK**	B08	1.030240705	0.653099509	57.74636028
**TTN**	O18	0.983121953	1.087397902	9.589493296
**TXK**	A20	1.127631343	1.011599992	11.47008223
**TXNDC3**	O20	1.030371633	1.66520337	38.12337572
**TXNDC6**	C05	0.858883684	0.585352932	46.72920157
**TYK2**	C20	0.576073991	1.142500227	49.57777885
**TYRO3**	D16	0.895507081	0.815771374	9.774271274
**UCK1**	A11	0.791091629	1.113487655	28.95371359
**UCK2**	I10	1.812273526	1.372761351	32.01664837
**UCKL1**	A12	1.138066104	1.197930456	4.997314471
**UHMK1**	M06	0.958356859	1.101165831	12.96888875
**ULK1**	G04	0.70418478	2.066911193	65.93057398
**ULK2**	M21	1.035084678	0.841236368	23.04326311
**ULK3**	O04	0.959672623	1.078207913	10.99373219
**ULK4**	C12	1.02578587	0.957575439	7.123243568

**VRK1**	E20	1.170383181	0.908343459	28.84808825
**VRK2**	F16	0.954874372	1.667152116	42.72422035
**VRK3**	M03	0.693365983	1.82248788	61.95497423
**WEE1**	G20	1.468168075	0.950080629	54.53089247
**WEE2**	I09	1.050636456	1.246456516	15.7101397
**WNK1**	D05	0.937009104	0.546957721	71.31289462
**WNK2**	P20	1.426663483	1.336228905	6.767895719
**WNK3**	J07	1.038328497	1.229131237	15.52338224
**WNK4**	A21	0.969403822	1.284121502	24.50840359
**XYLB**	J12	1.130391331	1.106116707	2.19458067
**YES1**	B14	0.947106634	1.068058233	11.32443858
**YSK4**	G10	0.722533127	0.894983889	19.26858846
**ZAK**	I07	0.665205891	2.004636161	66.81662718
**ZAP70**	K10	0.879721332	0.829146019	6.099687071
